# Clinical phenotype-based gene prioritization: an initial study using semantic similarity and the human phenotype ontology

**DOI:** 10.1186/1471-2105-15-248

**Published:** 2014-07-21

**Authors:** Aaron J Masino, Elizabeth T Dechene, Matthew C Dulik, Alisha Wilkens, Nancy B Spinner, Ian D Krantz, Jeffrey W Pennington, Peter N Robinson, Peter S White

**Affiliations:** Center for Biomedical Informatics, The Children’s Hospital of Philadelphia, Philadelphia, PA USA; Department of Pediatrics, The Children’s Hospital of Philadelphia, Philadelphia, PA USA; Department of Pathology and Laboratory Medicine, The Children’s Hospital of Philadelphia, Philadelphia, PA USA; Department of Pathology and Laboratory Medicine, University of Pennsylvania, Philadelphia, PA USA; Perelman School of Medicine, University of Pennsylvania, Philadelphia, PA USA; Institute for Medical Genetics and Human Genetics, Charité-Universitätsmedizin Berlin, Augustenburger Platz 1, 13353 Berlin, Germany; Berlin-Brandenburg Center for Regenerative Therapies, Charité-Universitätsmedizin Berlin, Augustenburger Platz 1, 13353 Berlin, Germany; Max Planck Institute for Molecular Genetics, Ihnestrasse 73, 14195 Berlin, Germany; Department of Pediatrics, Cincinnati Children’s Hospital and Medical Center, Cincinnati, OH USA; Department of Biomedical Informatics, University of Cincinnati College of Medicine, Cincinnati, OH USA

**Keywords:** Clinical, Phenotype, Exome, Genome, Informatics

## Abstract

**Background:**

Exome sequencing is a promising method for diagnosing patients with a complex phenotype. However, variant interpretation relative to patient phenotype can be challenging in some scenarios, particularly clinical assessment of rare complex phenotypes. Each patient’s sequence reveals many possibly damaging variants that must be individually assessed to establish clear association with patient phenotype. To assist interpretation, we implemented an algorithm that ranks a given set of genes relative to patient phenotype. The algorithm orders genes by the semantic similarity computed between phenotypic descriptors associated with each gene and those describing the patient. Phenotypic descriptor terms are taken from the Human Phenotype Ontology (HPO) and semantic similarity is derived from each term’s information content.

**Results:**

Model validation was performed via simulation and with clinical data. We simulated 33 Mendelian diseases with 100 patients per disease. We modeled clinical conditions by adding noise and imprecision, i.e. phenotypic terms unrelated to the disease and terms less specific than the actual disease terms. We ranked the causative gene against all 2488 HPO annotated genes. The median causative gene rank was 1 for the optimal and noise cases, 12 for the imprecision case, and 60 for the imprecision with noise case. Additionally, we examined a clinical cohort of subjects with hearing impairment. The disease gene median rank was 22. However, when also considering the patient’s exome data and filtering non-exomic and common variants, the median rank improved to 3.

**Conclusions:**

Semantic similarity can rank a causative gene highly within a gene list relative to patient phenotype characteristics, provided that imprecision is mitigated. The clinical case results suggest that phenotype rank combined with variant analysis provides significant improvement over the individual approaches. We expect that this combined prioritization approach may increase accuracy and decrease effort for clinical genetic diagnosis.

**Electronic supplementary material:**

The online version of this article (doi:10.1186/1471-2105-15-248) contains supplementary material, which is available to authorized users.

## Background

Thirty-five years since the introduction of contemporary DNA sequencing techniques, next generation sequencing (NGS) methods now enable rapid and inexpensive exome and whole genome sequencing [1–5]. Many genetic discovery applications such as population level mutation frequency analyses [6, 7] and cancer genomics [8, 9] have benefited from NGS capabilities. More recently, whole exome sequencing has played a clinical role for pediatric Mendelian disease and cancer diagnosis. Application to clinical rare disease diagnosis, however, remains challenging for many disorders with complex or multimodal genetic etiologies, primarily due to the difficulty of interpreting genomic sequence variants relevant to patient phenotypic features [10–13].

In addition to other factors, clinical interpretation of NGS results is difficult due to the scale and complexity of the test output data. Unlike typical clinical diagnostic tests that provide a small number of data points in well understood genes, a patient’s exome or genome usually contains hundreds or thousands of genetic variants, many of which require expert analysis to determine clinical relevance. Currently, each patient’s sequence reveals a number of predicted or possibly damaging variants that must be individually assessed for biological impact to gene function. However, variant impact to gene function alone is insufficient to determine clinical relevance. Clear association between a gene harboring a damaging variant and patient phenotype is also required for accurate clinical diagnosis. This gene-to-patient phenotype analysis is currently difficult for several reasons. First, there are tens of thousands of known gene-to-phenotype associations with various degrees of penetrance, which precludes individual expert assessment by a review process. Also, in many cases no single variant explains all phenotypic features present in a given patient, while several variants may explain a subset of the features. These complications typically require the involvement of a team of highly trained specialists to identify the variant(s) causative of patient phenotype, a process that is challenging with respect to expected future demand for clinical sequencing of cases with a complex genetic etiology [14, 15]. Analogous to the development of tools that aid variant biological impact assessment; similar methods for systematic phenotype analysis would likely assist this process [16, 17].

One approach to simplify NGS-aligned phenotype analysis is the use of gene lists. In this method, a predetermined set of genes with known or suspected pathogenicity for a diagnostic category (e.g., intellectual disability) is created, and variant analysis is restricted to this subset of candidate variants. In effect, the gene list is used as a phenotype filter applied prior to additional analysis. While this method provides some systematic phenotypic analysis, it has important drawbacks. It can be difficult to reach consensus on the number and breadth of phenotypic categories and the genes that should be included within a given category. Additionally, the lists require continual curation to remain current with new findings. Most importantly, the applied list acts as an inflexible filter that either strictly includes or excludes genes from further analysis.

A number of approaches have exploited prior gene knowledge, genomic, functional, and population structure features for variant prioritization and prediction of NGS data. Similarly, existing commercial tools, such as those offered by Cypher Genomics and Ingenuity, utilize proprietary algorithms to provide phenotype analysis for variant interpretation. However there is a need for continued research to develop improved, openly available methods that incorporate patient clinical phenotype for automated variant classification. Here, we implement and validate an algorithm that automatically ranks a gene set relative to provided phenotype descriptors. Algorithm performance is analyzed under a variety of scenarios intended to mimic clinical diagnostic conditions. The algorithm utilizes the concept of semantic similarity, which has been applied in a variety of biological studies [18–21]. The work presented in [21] used semantic similarity to rank candidate disease diagnoses relative to selected ontology terms describing patient phenotype. Our approach is similar but ranks candidate genes rather than diseases. Specifically, our model ranks genes by the semantic similarity between patient phenotype features and phenotype features directly associated with each of a patient’s mutated genes. The model validation results demonstrate that, in combination with the many diverse variant features currently considered by diagnostic laboratories, phenotypic similarity measures can provide value for variant prioritization in a clinical setting.

## Results and discussion

Once a patient’s exome or genome is sequenced, it is necessary to correlate a mutated gene to the patient’s phenotype to reach a clinical diagnosis. Diagnostic complexity may be reduced if the mutated genes can be automatically ranked relative to their relation to the patient’s phenotype. Here, we present simulation and clinical results for a semantic similarity algorithm that provides such a ranked list. For this exercise, we used the Human Phenotype Ontology (HPO), a controlled vocabulary organized as a strict hierarchy that largely represents clinical features and pathogenic variant data from the Online Mendelian Inheritance in Man (OMIM) resource and the biomedical literature.

**Figure 1:**
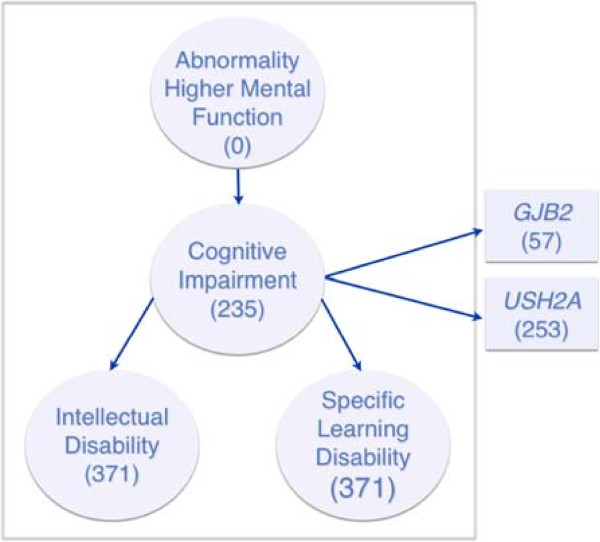
**Subsection of human phenotype ontology (HPO).** Terms at left illustrate the ontology’s hierarchical arrangement. The numbers in parentheses indicate the number of genes that term directly annotates. A term is considered to annotate a gene if it directly annotates the gene, as shown in figure, or if it indirectly annotates the gene through any of its descendants, e.g. “Abnormality Higher Mental Function” is considered to annotate all 235 genes annotated by “Cognitive Impairment”. The gene list to the right illustrates how HPO terms are annotated to genes. The numbers in parentheses indicate the total number of HPO terms that directly annotate the gene.

Our algorithm computes a score for a given gene by comparing the similarity of HPO terms annotated to the gene and those used to describe the patient. The similarity of any two HPO terms is a function of the specificity of the two terms and their semantic relation. The specificity of a term is quantified by its information content, *IC,* which is a function of the number of genes it annotates (see Equation 1). There is an inverse relation between *IC* and the number of annotated genes, i.e. the more genes a term annotates, the lower the term’s information content. In this study, the similarity of two HPO terms is defined as the *IC* of the most informative common ancestor of the two terms (see Equation 2). For example, the similarity of the terms “*Intellectual Disability”* and *“Specific Learning Disability”* is the IC of the term *“Cognitive Impairment”* as illustrated in Figure 1. The maximum similarity between each patient term and each gene annotation averaged over the number of patient terms is the similarity score for the gene (see Equation 3). These scores, with higher scores indicating a stronger predicted relation to the patient phenotype, can be used directly to rank genes.

### Analysis

We assessed algorithm performance by the ability to rank the known causative gene highly within a given gene list. In each case, we computed the semantic similarity score between the patient’s phenotype terms and the phenotype terms associated with the genes in the gene list. We then sorted the gene list relative to the computed similarity scores and identified the causative gene’s rank. Additionally, we associated a p-value with each observed score to account for annotation bias that can occur when objects are annotated with ontology terms. Bias can result from differences in curation and because some diseases—and by extension the related gene—have more phenotypic features, e.g. non-syndromic hearing loss vs. neurofibromatosis type 1. Compared to a given query term set, the term set of a preferentially annotated object is more likely by random chance to have a higher semantic similarity score than the term sets of other, less annotated objects. Consequently, the ordering of an object set by comparison of the semantic similarity score of each object’s annotation set to the query set can be skewed. To compensate for annotation bias, we considered the one-sided p-value associated with an observed semantic similarity score. As with the similarity scores, we sorted the gene list relative to the p-values and identified the causative gene’s rank.

### Simulation cases

**Figure 2:**
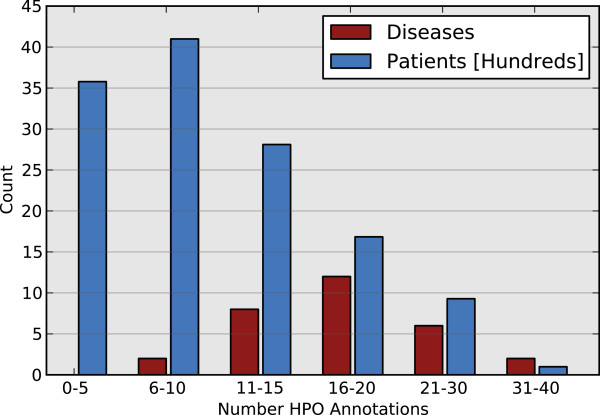
**Simulated HPO term annotation counts.** Distribution of the number of HPO term annotations per disease for the 33 Mendelian diseases used in the simulation cases (shown in red), and the number of HPO term annotations per simulated patient (shown in blue).

Simulated results were generated for 33 diseases that have a single known causative gene according to the OMIM database and for which sufficient phenotype feature penetrance data were available to accurately model patient characteristics [21, 22]. For these 33 diseases, the number of HPO annotations per disease is approximately normally distributed, with a range from 6 to 50 and a mean of 19.7 (Figure 2). An additional file lists the diseases and their associated HPO terms and penetrance [see Additional file 1]. For each disease, we first generated 100 simulated patients by selecting HPO terms for the patient from those terms directly annotated to the disease gene, with probability determined by the penetrance data (see Methods). This case is considered optimal and represents a clinical scenario where the patient phenotype is well recognized and a specific causative gene is suspected. We then added *noise* terms (terms unrelated to the causative gene) to simulate clinical scenarios where certain patient characteristics are unrelated to the disease. For each patient, the number of noise terms was taken to be half the number of optimal terms, e.g. if a patient had 10 optimal terms, 5 randomly selected noise terms were added. Finally, we considered *imprecision*, which occurs when selected patient terms are related to the disease but are less specific than the terms annotated to the causative gene. Imprecision was simulated by randomly replacing each optimal patient term with one of its ancestor terms. In all, we generated 13,200 simulated patients with a range of HPO annotations between 1 and 39 and a mean of 9.4 (Figure 2).

**Figure 3:**
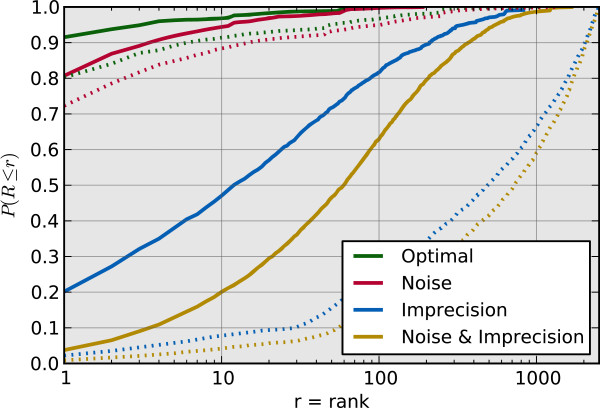
**Causative gene rank cumulative distribution function.** The cumulative distribution function of causative gene rank for the four simulated scenarios taken across the 33 simulated diseases. The solid lines are the results obtained when ranked by similarity score. The dashed lines are the results obtained when ranked by p-value. The *x-axis* is the rank, *r*, and the *y-axis* is the probability that the causative gene rank, *R*, is less than or equal to *r*. Note that the *x-axis* is on a logarithmic scale.

For each simulated patient, we ranked the causative gene against all 2488 genes annotated by at least one HPO term to evaluate algorithm performance. The causative gene rank cumulative distribution plots, shown in Figure 3, summarize the results. The causative gene was ranked first for 92% of the optimal cases when ranked by similarity score and 80% when ranked by p-value. The occurrence of optimal cases with causative gene ranks other than first is a result of the patient phenotype annotation process. Recall that optimal patient terms were selected probabilistically based on term penetrance. Thus some optimal patients had fewer annotations and/or were not annotated with the important disease terms, specifically those with higher information content.

The addition of noise alone did not have substantial impact on the rankings. When introducing noise, the causative gene was ranked first for 80.6% of the cases when ranked by similarity score and 72% when ranked by p-value. Further, the causative gene was ranked in the top 10 for greater than 88% of the cases when ranked by similarity score or p-value.

**Figure 4:**
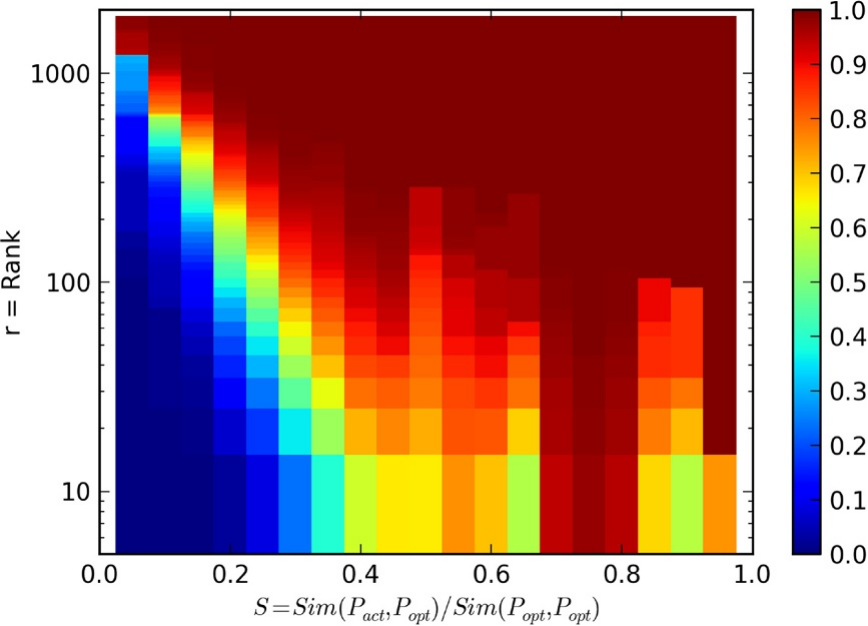
**Noise and imprecision quantification.** The color map indicates the probability that the actual causative gene rank, *R*, is less than or equal to *r (y-axis)* given the similarity value, *S (x-axis)*, between the actual and optimal patient, that is the similarity between the patient terms with noise and/or imprecision, *P*_*act*_, and the corresponding optimal patient terms, *P*_*opt*_. Similarity values are normalized by the self-similarity between the optimal patient terms so that all values of S are in [0,1].

However, we determined that introducing imprecision had a large impact. The causative gene was ranked first for 20% of the cases with added imprecise terms when ranked by similarity score, and 2% when ranked by p-value. When we considered introduction of both imprecision and noise, these values fell to 4% and held at 2% when ranked by similarity score and p-value, respectively. However, these results are skewed by outliers that occur for patients with significant amounts of noise and imprecision. This effect is observed in Figure 4, which relates the quantity of noise and imprecision in the patient terms to causative gene rank. Specifically, Figure 4 illustrates the probability that the causative gene is ranked above a certain value as a function of the similarity between the optimal patient terms and the corresponding patient terms with noise and/or imprecision when ranked by similarity score. The figure indicates that when this similarity is greater than 0.5—when the noise and imprecision terms are not too severe—the causative gene is ranked in the top 20 genes for at least 80% of cases. Indeed, the median causative gene ranks were 12 and 60 respectively for cases with imprecision, and imprecision with noise cases when ranked by similarity score. Thus, for the majority of cases, the causative gene was ranked in the top 2.5%. Note that the maximum probability observed for *S* between 0.7 and 0.8 is likely a result of the specific random patients generated in this study. Given more samples, we expect that the maximum would shift toward *S =* 1.0. However, the general trend observed in the plot of decreasing rank (increasing performance) with increasing *S* would be maintained with more samples and is indicative of the algorithm’s performance relative to imprecision and noise.

There is some indication that inclusion of patient exome or whole genome data can further improve results under these conditions. As described in the clinical results section, patient NGS data can be used to filter predicted non-damaging variants and common variants, which reduces the gene list length. Additionally, the reduced gene list can be used to guide patient phenotype feature selection, a topic we discuss further below.

**Figure 5:**
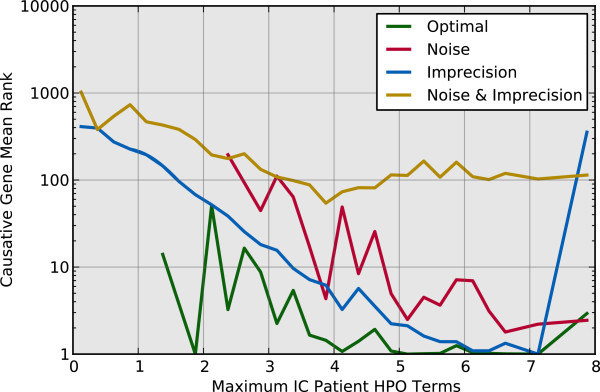
**Patient HPO annotation information content impact.** The causative gene mean rank when ranked by similarity score as a function of the maximum information content, IC, of all HPO terms used to describe the patient. Higher information content indicates higher term specificity.

**Figure 6:**
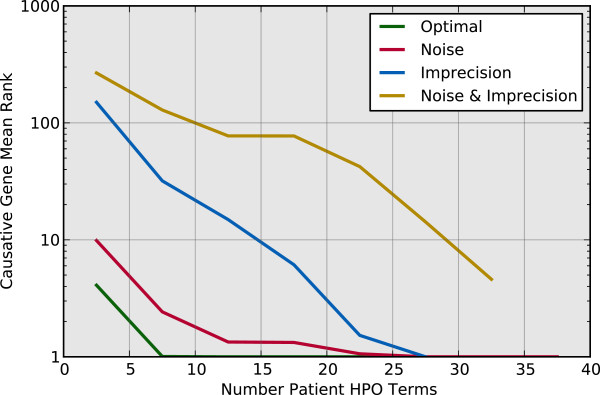
**Patient HPO annotation count impact.** The causative gene mean rank when ranked by similarity score as a function of the number of HPO terms used to describe the patient.

The noise and imprecision analysis on algorithm performance suggests possible guidelines for selecting patient terms in clinical practice. In particular, it appears that the algorithm performs better when provided patient terms that are very specific, and by having a large number of relevant terms (Figures 5 and 6). Comparison of the noise-with-imprecision case to the noise-only and imprecision-only cases in Figure 5 indicates that selecting very specific terms can help counter noise. As with Figure 4, the minimum rank (best performance) that occurs as the maximum IC reaches 7 in Figure 5 is likely a result of the specific random sample observed in this study. Given more samples, we expect that the best performance would shift toward a maximum IC of 8, which is the maximum possible for the particular annotation set used. Again, we expect that the general trend observed in the plot of decreasing rank (increasing performance) with increasing maximum IC would be maintained with more samples. Figure 6 indicates that both imprecision and noise effects can be countered by selecting a large set of patient terms, provided that the terms are relevant to the disease condition.

### Clinical cases

To provisionally evaluate the algorithm in a clinical workflow, we measured performance for four actual clinical cases with hearing impairment. A single genetic counselor provided clinically relevant patient phenotypes for each of the four subjects as part of a retrospective exome sequencing validation study. Notably, for more complex phenotypes it is likely that multiple clinicians may collectively select patient phenotype terms. In such cases, variability in term selection between clinicians is possible and may increase imprecision. The counselor was allowed to select any HPO phenotype term deemed clinically relevant, i.e. term-to-gene annotation information was not provided as might be done to mitigate noise and imprecision. Terms were selected for a given patient using a custom web application that enables HPO term searches by integration with NCBO web services. The web application associates the selected terms with a patient ID and stores the terms for later use. Semantic similarity scores were determined for each case prior to considering genomic sequence data. Subsequently, exome sequence data was obtained for each case. Similarity scores were then recalculated considering only the subset of genes that contained one or more infrequent or rare variants in each individual patient.

Table 1 provides causative gene ranks for the four hearing impaired subjects. The patient annotations ranged from 2 to 6 HPO terms. When compared to all 2488 HPO annotated genes, the causative gene exhibited a mean rank of 22.25 (range: 1–44) when ranked by similarity score and 76.5 (range: 24–218) when ranked by p-value.

**Table 1:** **Clinical cases causative gene rank**

***Gene***	***Terms***	***HPO sim score rank***	***HPO p-value rank***	***Variant gene set***	***Gene set sim score rank***	***Gene set p-value rank***
*USH2A*	2	12	38	113	2	1
*CDH23*	6	32	24	125	4	3
*SLC26A4*	4	1	26	135	1	1
*GJB2*	3	44	218	129	4	16

Causative gene ranks for patients with hearing impairment. Gene: the causative gene for each case. Terms: the number of HPO phenotype terms assigned to the subject by the genetic counselor. HPO Sim Score Rank: the calculated causative gene rank using the semantic similarity score compared to all 2,488 HPO annotated genes. HPO p-value Rank: the calculated causative gene rank using the p-value compared to all 2,488 HPO annotated genes. Variant Gene Set: the number of genes with variants identified in the patient of interest. The variant gene set represents genes for which the patient had exonic or splice site variants that occur with a minor allele frequency: <10% in CHOP’s internal cohort or <3% in exomes from either the 1000 Genomes Project [23] or the NHLBI Exome Variant Server [24]. Gene Set Sim Score Rank: the calculated causative gene rank using the semantic similarity score when compared to the subset of genes included in that patient’s variant gene set. Gene Set p-value Rank: the calculated causative gene rank using the p-value when compared to the subset of genes included in that patient’s variant gene set.

After consideration of exome data for each patient and filtering out non-exomic and common variants, each patient contained variants in approximately 125 genes (mean: 125.5; range 113–135). When re-ranking within this subset of variant-associated genes, the causative gene demonstrated a mean rank of 2.75 (range: 1–4) when ranked by similarity score and 5.25 (range: 1–16) when ranked by p-value. Although preliminary, this result implies that combining phenotypic information via the semantic similarity score with population and structural information may provide a significant improvement in the ability to identify patient causative variants in certain patient populations.

As noted, our method is similar to the tool presented in [21], *Phenomizer,* which ranks candidate diseases relative to patient phenotype. Where possible, the *Phenomizer* tool also provides genes known to be associated with each disease, thereby providing an indirect gene rank mechanism. However, the resulting *Phenomizer* gene rank is based on *HPO term-to-disease* annotations whereas the method presented here provides gene ranks based on *HPO term-to-gene* annotations. These two annotation sources, term-to-disease and term-to-gene, have different distributions. The annotation differences arise because many of the annotated diseases lack specific gene associations and many diseases are associated with multiple genes that individually are not associated with all of the phenotype characteristics associated with the disease. Consequently, the two methods yield different gene rank results. For example, for the four clinical cases presented here, *Phenomizer* yields mean causative gene ranks of 29.25 and 111.75 out of the 2488 annotated genes when ranked by similarity score and p-value, respectively. Selection of one approach over the other depends on the clinical scenario. However, in cases where whole exome or whole genome sequencing is performed and information specific to gene rank is sought, the presented method is likely advantageous, as it is based on direct phenotype term-to-gene knowledge and appears to rank the causative gene more highly.

## Conclusion

These results suggest that semantic similarity can rank a causative gene highly within a gene list relative to patient phenotype characteristics. The algorithm demonstrates good performance in the presence of noise, provided that imprecision is mitigated. Further, the clinical case results suggest that phenotype rank combined with variant analysis can substantially improve results over the individual approaches, at least within the diagnosis of hearing impairment. These conditions approximate a typical rare disease diagnosis analysis scenario that exists after a patient’s sequence results are complete and a set of genes with identified variants is produced. In this case, noise and imprecision can be mitigated if patient phenotype term selection is restricted to a subset of HPO terms, namely the terms annotated to the patient’s variant genes.

While the clinical application has been applied here only to a small number of cases associated with a single diagnostic category, these results are encouraging, as they suggest that careful annotation of phenotype by clinical staff can dramatically improve gene rankings, and thus could significantly reduce analysis time in certain rare disease cases, when assessed in context with additional genomic and clinical data. However, careful annotation of cases in a busy clinical setting presents workflow and expertise resourcing challenges. To mitigate these obstacles, we are currently developing software that will assist patient phenotype selection through use of the known term information content and intelligent term suggestion.

As compared to phenotype-based gene lists, the semantic similarity-ranked patient gene list provides at least two advantages. First, the relative order provides more information in the situation where the patient’s mutated gene set contains multiple genes from the assigned gene list. In this situation, a gene list does not prioritize amongst multiple genes, and hence all must be investigated equally. The rank ordered list, however, assigns a score to each gene that indicates the strength of association between the gene and the patient’s clinical presentation. Genes with very low scores are unlikely to be associated with the patient’s specific phenotype and should not require detailed review. If we assume there is a reliable mechanism to determine a similarity score or p-value threshold to delineate genes unassociated with patient phenotype, we may expect reduced analysis duration. Secondly, unlike a phenotype-specific gene list, the rank ordered list is inclusive of all annotated genes, not just those on the phenotype specific gene list, which may not be comprehensive for the phenotype. Indeed, this was encountered in a hearing impairment subject whose causative mutation occurred within the *CDH23* gene. A team of physicians and genetic counselors with pediatric hearing loss expertise created a hearing impairment gene list for use at The Children’s Hospital of Philadelphia. The hearing impairment gene list is available as an additional file [Additional file 2]. The list was created to support WES clinical analysis conducted for ongoing research within the National Institutes of Health (NIH) Clinical Sequencing Exploratory Research (CSER) consortium and was used in the initial analysis for the cited case. However, the gene list did not include *CDH23* and the diagnosis was missed. In contrast, our semantic similarity algorithm ranked the *CDH23* gene 2^nd^ among the 125 genes with variants of interest. This is an anecdotal case but it illustrates the challenge with curating phenotype based gene lists.

An important algorithm limitation is that it is knowledge-based and is therefore limited by the gene annotation sources being considered. As such, the system cannot rank any gene that is not annotated by at least one HPO term and is dependent upon available gene-to-HPO term mappings. In such cases, the unranked genes should be clearly delineated. Although we focused initially on HPO, we have designed the algorithm to easily consider additional disease-based ontologies and expect that more comprehensive vocabularies and term-to-gene mappings will emerge over time. Another limitation is that the algorithm does not consider pertinent negative annotations, i.e. the indication that relevant phenotypic terms are not present in the patient. Inclusion of negation information could be valuable but may likely require better disease term penetrance data to develop adequate scoring models.

Currently, good algorithm performance is constrained to the clinical diagnostic scenario described above in order to mitigate imprecision. If imprecision degradation can be reduced or eliminated (e.g., when applied to disorders with strong genotype-to-phenotype correlations), algorithm applicability may expand to other scenarios, such as pre-test decision support for appropriate genetic test selection. A first step in this regard is to develop high-resolution similarity score distribution models to obtain accurate p-values that can serve as a test validation measure. For example, assume the top ranked gene has a similarity score, *s*, with an associated p-value, *α*. The absolute similarity score, *s*, only provides an inter-gene comparison mechanism but does not indicate the statistical significance of the relation between the top ranked gene and the patient phenotype. A p-value would provide such a statistical significance measure. Note, in this context it is necessary to correct for multiple testing [25]. We are currently developing similarity score distribution models with higher resolution than our initial versions that will enable reliable p-value calculations to indicate test validity.

Exome sequencing capabilities have enabled advancements in a number of genetic research domains, clinical diagnosis of Mendelian disease, and clinical oncology. However, application of genomic sequencing as a clinical diagnostic test has been more difficult to achieve for many rare diseases. In part, this difficulty results from the complicated process of associating predicted deleterious variants to observed patient phenotype. Moreover, clinical application of NGS technologies to rare disease must often address a wide phenotype spectrum. Specific phenotype combinations will vary widely in their ability to precisely define genes. For example, the search query “microcephaly, oligodactyly, diaphragmatic hernia, hypertrichosis” produces exactly one OMIM result, Cornelia De Lange Syndrome. Other phenotypes are more difficult to diagnose because they are common and exhibit genetic heterogeneity. Our clinical results are encouraging because the hearing disorder phenotype is especially difficult to diagnose; indeed, the search query “hearing loss” yields greater than 1000 OMIM results. With continued research, we expect the algorithm presented here may play an integral role for NGS-based clinical diagnosis, especially when used in combination with other genomic, population, and predictive variant annotations.

## Methods

Development of our prioritization model required a controlled vocabulary to describe phenotypes and knowledge of the semantic relationships between the vocabulary terms. Additionally, the genes that are to be ranked must be annotated with the appropriate vocabulary terms. The first two requirements are satisfied using an ontology that includes a shared vocabulary and defined relationships between vocabulary terms. Appropriate annotation of ontology terms to genes satisfies the last requirement. For the work presented here, we used the Human Phenotype Ontology (HPO) [26–28], which was specifically developed to represent human disease phenotypic abnormality knowledge. HPO concepts are organized in a strict hierarchy, where child terms assume “is-a” relationships with their ancestor terms. In addition to the ontology, the HPO resources include a gene-to-phenotype mapping that provides known associations between Entrez gene IDs [29, 30] and HPO terms. The true-path rule applies to all annotations, such that if a gene is annotated with a specific HPO term, it inherits all ancestors of that term. The specific files used for this work were hp.obo build 687, and genes-to-phenotype build 26. This HPO version contains 9,965 phenotype terms, of which 6,346 either directly or indirectly annotate at least one gene. The genes-to-phenotype file provides 61,784 direct annotations for 2,488 genes.

### Semantic similarity

Our model is based on a concept known as semantic similarity. We assume that ontology terms are used to annotate a set of objects, and that the true-path rule applies for all term assignments. In this case, a given term will be more or less specific than others with respect to the number of objects it annotates. Consider two terms sets, *P*_*1*_*= {p*_*1*_*,p*_*2*_*,…,p*_*K*_*}* and *P*_*2*_*= {p*_*1*_*,p*_*2*_*,…,p*_*J*_*}*, selected from the ontology. Given the semantic structure of the ontology and the specificity of the terms, we can compare the similarity, or likeness, of these sets with an appropriate semantic similarity model.

Our semantic similarity model requires a mathematical description of each ontology term’s specificity. Given an ontology term, *p*, we quantify the term’s specificity using its information content, *IC*, as defined by Resnik [31]:
1

where *N* is the total number of objects annotated by all terms and *n*_*p*_ is the number of objects annotated either directly or indirectly, via the true-path rule, by the term *p*. Given two terms *p*_*1*_ and *p*_*2*_, let *A(p*_*1*_*,p*_*2*_*)* be the set of all common ontological ancestors of *p*_*1*_ and *p*_*2*_, noting that *p*_*1*_ and *p*_*2*_ are not included in *A* unless *p*_*1*_*= p*_*2*_. We define the most informative common ancestor of *p*_*1*_ and *p*_*2*_, *p*_*MICA*_, to be the term in *A* with the maximum information content. We define the termwise semantic similarity as [21]:
2

where *n*_*pMICA*_ is the number of objects annotated directly or indirectly by the term *p*_*MICA*_.

In order to compare two term sets, we require a term-set semantic similarity model. Given two arbitrary term sets, *P*_*1*_*= {p*_*1*_*,p*_*2*_*,…,p*_*K*_*}* and *P*_*2*_*= {p*_*1*_*,p*_*2*_*,…,p*_*J*_*}*, we define the asymmetric term-set semantic similarity as:
3

where *sim*_*X*_ is a termwise similarity function such as Equation 2. Note, Equation 3 is asymmetric, i.e., its output is dependent on the order of the arguments *P*_*1*_ and *P*_*2*_. A symmetric term-set semantic similarity model can then be constructed as follows:
4

Note that Equations 3 and 4 describe a family of semantic similarity models that depend on the termwise comparison function *sim*_*X*_, a number of which are described in the literature [21, 31, 32].

Given a term-set semantic similarity model, we can rank an annotated object set relative to some query set. Here, we wish to rank a list of genes relative to a query set that represents the patient’s phenotype. We first assume that a set of HPO phenotype terms, *P*_*p*_*= {p*_*1*_*,p*_*2*_*,…,p*_*K*_*}*, is used to characterize patient abnormalities, and that any given gene, *g*, is annotated with a set of phenotype terms, *P*_*g*_*= {p*_*1*_*, p*_*2*_*, . . . , p*_*J*_*}*, that indicate a known link between mutations in the gene and the associated phenotype abnormalities. For each gene, we assign a score computed as the semantic similarity between *P*_*p*_ and *P*_*g*_ and then sort the genes in descending order based on the assigned score. In the event of identical values across multiple genes, all equivalent genes are assigned a rank equal to the mean of the sequential positions. For example, if four genes all have the same similarity score and the four genes occupy positions *{i, i* + *1, i* + *2, i* + *3}* in the sorted list, then each gene is assigned the rank *(4i* + *6)/4*, rounded down to the nearest integer.

### Statistical significance

To compensate for annotation bias, objects can be ordered by some statistical significance measure of the semantic similarity observed between the query term set and the terms annotated to each object. Specifically, we computed the one-sided p-value associated with an observed semantic similarity score. A similarity score distribution model is required for each annotated object and for each fixed query term set length to determine the p-value of an observed similarity score. We consider two objects, *g*_*1*_ and *g*_*2*_, each annotated with different ontology terms, assuming *K*_*1*_ ontology terms are randomly selected to represent the query set and that the semantic similarity between the query set and the terms annotated to *g*_*1*_ and *g*_*2*_ are recorded. The score distribution observed for *g*_*1*_ will differ from that observed for *g*_*2*_. Similarly, the distribution obtained for *g*_*1*_ with randomly selected query sets with *K*_*2*_ terms will differ from the distribution for *g*_*1*_ with query sets with *K*_*1*_ terms provided *K*_*2*_ *≠ K*_*1*_.

Assuming we have similarity score distribution models for each gene annotated by HPO terms for each phenotype query set length, *K*, and given an HPO phenotype query set representing a patient’s phenotypes, we can compute a semantic similarity score for each gene in some gene set as described in the previous section. If we now assign to each gene the p-value associated with the semantic similarity score observed for that gene, we rank the genes in ascending order by p-value. If two or more genes have the same p-value, their relative rank is determined using the similarity scores. For example, if four genes have the same p-value and occupy positions *{i, i* + *1, i* + *2, i* + *3}* in the sorted list, each gene is assigned the rank *i* - *1* + *s*_*r*_ where *s*_*r*_ is the gene’s position amongst the four genes when sorted by similarity score.

It is not computationally feasible to sample the entire similarity score space for all genes annotated by HPO terms because the number of possible query phenotype sets grows exponentially with query set length, *K*. Consequently, we estimated the similarity distribution models using Monte Carlo sampling. For each combination of gene and query set length in the range [2, 10], we randomly selected 100,000 query phenotype sets and computed the corresponding semantic similarity to the terms annotated to the gene. Note that for *k = 1*, we sampled the entire space of 6,346 query terms. The Monte Carlo sampling was conducted using a custom Scala Akka application deployed on Amazon’s Elastic Compute Cloud [33–35].

From the Monte Carlo samples, we created tables representing the similarity score cumulative distribution function (*CDF*) for each gene and patient phenotype set length combination. Given a similarity score, *s*, for gene, *g*, and patient phenotype set length, *K*, we estimate the one-sided p-value for the probability of randomly obtaining a similarity score greater than or equal to the observed score from the *CDF* as:
5

For query phenotype sets of length greater than 10, we used the tables derived for *K = 10*.

### Data generation

For a given disease, we formed a baseline disease phenotype feature set as the intersection of the disease terms supplied in the online version of [21] and those annotated to the Online Mendelian Inheritance in Man (OMIM)-indicated causative gene, per the HPO resource file gene-to-phenotypes. The penetrance of each disease phenotype was also taken from the online version of [21]. A complete list of the disease causative genes and their associated phenotypes is available as an additional file [see Additional file 1].

We simulated 100 baseline patients per disease. To account for gender-specific phenotypes, we first generated a standard uniform random number and designated the patient as male if the number was greater than 0.5, and female if ≤0.5. Then, for each baseline phenotype feature, *p*, associated with the patient’s disease, we generated a new standard uniform pseudo-random number, *α*_*p*_, and added *p* to the patient’s phenotype set if *α*_*p*_ *≤ f*_*p*_, where *f*_*p*_ is the phenotype penetrance. For example, assume a given disease has two phenotypes, *p*_*1*_ and *p*_*2*_, with penetrance 0.25 and 0.5, respectively, then for each simulated patient we generated two random numbers, *α*_*p1*_ and *α*_*p2*_ . We assigned *p*_*1*_ to the patient if *α*_*p1*_ *≤ 0.25* and likewise assigned *p*_*2*_ if *α*_*p2*_ *≤ 0.5*. We tacitly assumed that disease phenotypes occur independently of each other because joint distribution data is not currently available. We ensured that every simulated patient had at least one phenotype term. We denote this patient cohort as “optimal” to indicate that the patient phenotype characteristics were selected from those directly annotated to the causative gene.

In actual clinical practice, patient phenotype sets will often include noise and imprecision. Imprecision occurs when a less specific phenotype term is used to annotate the patient in place of the phenotype term annotated to the causative gene. For example, assume the phenotype, *p*_*1*_, is directly annotated to the causative gene, *g*. Let *p*_*2*_ be any ontological ancestor of *p*_*1*_ other than the ontology root term, and assume that *p*_*2*_ is not annotated to *g*. We defined imprecision as present if *p*_*2*_ is used to annotate the patient instead of *p*_*1*_. We defined noise as occurring when a phenotype that does not directly or indirectly annotate a gene is nevertheless assigned to the patient phenotype set.

To examine the impact of noise, randomly selected noise terms were added to each patient’s baseline optimal phenotype set. Noise terms were generated separately for each patient. Each disease was associated with an allowable set of HPO noise terms, namely all terms that do not directly or indirectly annotate the disease causative gene. For each patient, noise terms were selected randomly from the disease specific list so that different patients had different noise terms. The number of noise terms per patient was set to half the number of optimal terms, i.e. those derived from terms annotating the causative gene. For example, if a given patient had 4 optimal terms that patient was assigned 2 additional randomly selected noise terms. If a given patient had 10 optimal terms that patient was assigned 5 additional randomly selected noise terms. To examine the impact of imprecision, the patient’s optimal phenotypes were each replaced with a randomly selected ancestor term, other than the ontology root. For each simulated disease, the terms directly annotated to the causative gene, i.e. the optimal terms, were assigned an allowed imprecision term set composed of all ancestor terms of the optimal term. For each patient, imprecision was added by replacing every optimal term by a randomly selected term from the imprecision set for that optimal term so that different patients had different imprecision terms. Finally, to add both noise and imprecision, the noise terms generated for a given patient in the “noise only” case were added to the patient terms generated in the “imprecision only” case.

In addition to the simulated data, we also obtained data for actual clinical patients taken from a research cohort currently used as part of a clinical genomics workflow validation project at The Children’s Hospital of Philadelphia and the Perelman School of Medicine at the University of Pennsylvania. The purpose of this project is to validate the ability of the exome sequencing workflow to enable the identification of causative variants. The cohort subjects all have known genetic diseases with a previously identified causative variant confirmed by Sanger sequencing. In the validation study, retrospective exome sequencing was performed for each subject. Genetic counselors, who were not provided the known causative variant, then examined the exome sequence results to identify the causative variant. For a number of these retrospective cases, a genetic counselor provided a list of patient HPO phenotype characteristics deemed relevant to the patient’s condition, which were used to evaluate the semantic similarity algorithm. This research was approved by the Institutional Research Board of The Children's Hospital of Philadelphia. Written informed consent was obtained from the patient for the publication of this report and any accompanying images.

### Data access

Two additional files accompany this article. “Additional file 1” is a .pdf file that contains the OMIM disease ID, name, causative gene, associated HPO terms and penetrance values used for the 33 simulated Mendelian diseases used in this study. “Additional file 2” is a .txt file containing the hearing loss phenotype gene list developed at The Children’s Hospital of Philadelphia and used for evaluation of the clinical cases in this study. The custom source code used to implement the algorithm and generate the simulated data in this work is publically available at: https://github.com/cbmi/phenomantics.

## Electronic supplementary material

Additional file 1: **Simulated Mendelian Disorders.** (PDF 252 KB)

Additional file 2: **Hearing Disorder Gene List.** (TXT 1 KB)
