# Supplementary material for: Clinical phenotype-based gene prioritization: an initial study using semantic similarity and the human phenotype ontology
Source: BMC Bioinformatics. 2014 Jul 21;15(1):248. doi: 10.1186/1471-2105-15-248 (PMC4117966; doi:10.1186/1471-2105-15-248)
Supplement: Supplementary file 1 — Additional file 1: Simulated Mendelian Disorders. (PDF 252 KB) [file 12859_2014_6518_MOESM1_ESM.pdf]

Appendix Simulated Diseases

OMIM: Disease ID: 101000, disease name: neurofibromatosis, type ii, causative gene: NF2

HPO Term ID	Gender	Penetrance
HP:0009589	N/A	0.85
HP:0007935	N/A	0.72
HP:0009593	N/A	0.68
HP:0002858	N/A	0.45
HP:0007876	N/A	0.41
HP:0009595	N/A	0.27
HP:0000360	N/A	0.1
HP:0009594	N/A	0.09
HP:0001251	N/A	0.08
HP:0009590	N/A	0.06
HP:0009592	N/A	0.04
HP:0002888	N/A	0.025

OMIM: Disease ID: 107480, disease name: townes-brocks syndrome, causative gene: SALL1

HPO Term ID	Gender	Penetrance
HP:0001177	N/A	0.5
HP:0001199	N/A	0.5
HP:0000384	N/A	0.5
HP:0001864	N/A	0.5
HP:0002023	N/A	0.4696
HP:0000003	N/A	0.075
HP:0000083	N/A	0.075
HP:0000110	N/A	0.075
HP:0000089	N/A	0.075
HP:0000076	N/A	0.075

OMIM: Disease ID: 108120, disease name: arthrogryposis, distal, type 1a, causative gene: TPM2

HPO Term ID	Gender	Penetrance
HP:0001838	N/A	0.88
HP:0001374	N/A	0.38
HP:0003184	N/A	0.38
HP:0003273	N/A	0.38
HP:0001848	N/A	0.33
HP:0001188	N/A	0.25
HP:0001762	N/A	0.25
HP:0001181	N/A	0.25
HP:0001032	N/A	0.25
HP:0009742	N/A	0.17
HP:0000954	N/A	0.075
HP:0000211	N/A	0.075
HP:0000028	N/A	0.075

OMIM: Disease ID: 113620, disease name: branchiooculofacial syndrome, causative gene: TFAP2A

HPO Term ID	Gender	Penetrance
HP:0002162	N/A	0.92
HP:0000368	N/A	0.9047
HP:0000579	N/A	0.7435
HP:0000470	N/A	0.66
HP:0002987	N/A	0.64
HP:0000506	N/A	0.58
HP:0001057	N/A	0.57
HP:0000987	N/A	0.57
HP:0000545	N/A	0.57
HP:0000508	N/A	0.57
HP:0000639	N/A	0.57
HP:0000945	N/A	0.51
HP:0000582	N/A	0.48
HP:0000568	N/A	0.44
HP:0000528	N/A	0.44
HP:0008559	N/A	0.43
HP:0001256	N/A	0.4166
HP:0000405	N/A	0.3684
HP:0000047	M	0.33
HP:0000486	N/A	0.3055
HP:0002020	N/A	0.3
HP:0000545	N/A	0.2812
HP:0000508	N/A	0.2777
HP:0000518	N/A	0.2424
HP:0002216	N/A	0.2368
HP:0000268	N/A	0.2307
HP:0001250	N/A	0.23
HP:0008606	N/A	0.15
HP:0000407	N/A	0.075

HP:0000104	N/A	0.075
HP:0001177	N/A	0.075
HP:0000196	N/A	0.075
HP:0002335	N/A	0.075
HP:0002211	N/A	0.075
HP:0000455	N/A	0.075
HP:0000175	N/A	0.075
HP:0000252	N/A	0.075
HP:0002020	N/A	0.075
HP:0005217	N/A	0.075
HP:0004785	N/A	0.075
HP:0002021	N/A	0.075
HP:0004464	N/A	0.075

OMIM: Disease ID: 113650, disease name: branchiootorenal syndrome, causative gene: EYA1

HPO Term ID	Gender	Penetrance
HP:0009795	N/A	0.5
HP:0009796	N/A	0.5
HP:0000378	N/A	0.45
HP:0000402	N/A	0.3
HP:0000799	N/A	0.1
HP:0000691	N/A	0.075
HP:0009798	N/A	0.075
HP:0000384	N/A	0.075
HP:0000175	N/A	0.075
HP:0000156	N/A	0.075
HP:0000193	N/A	0.075
HP:0002060	N/A	0.075
HP:0009797	N/A	0.075
HP:0001374	N/A	0.075

OMIM: Disease ID: 119500, disease name: popliteal pterygium syndrome, causative gene: IRF6

HPO Term ID	Gender	Penetrance
HP:0000175	N/A	0.9298
HP:0009756	N/A	0.5789
HP:0000196	N/A	0.4561
HP:0009754	N/A	0.4035
HP:0000046	M	0.3684
HP:0000048	M	0.3684
HP:0000028	M	0.3684
HP:0000013	F	0.3684
HP:0000726	F	0.3684
HP:0000059	F	0.3684
HP:0009758	N/A	0.3333
HP:0009755	N/A	0.1754
HP:0001762	N/A	0.1403
HP:0009757	N/A	0.0877
HP:0003298	N/A	0.075

OMIM: Disease ID: 121050, disease name: arthrogryposis, causative gene: FBN2

HPO Term ID	Gender	Penetrance
HP:0001519	N/A	0.86
HP:0001166	N/A	0.86
HP:0002987	N/A	0.86
HP:0008962	N/A	0.65
HP:0001762	N/A	0.32
HP:0000248	N/A	0.29
HP:0000268	N/A	0.29
HP:0003273	N/A	0.26
HP:0001653	N/A	0.25
HP:0001840	N/A	0.25
HP:0000545	N/A	0.075
HP:0002616	N/A	0.075
HP:0001631	N/A	0.075
HP:0001634	N/A	0.075
HP:0001629	N/A	0.075
HP:0002999	N/A	0.075

OMIM: Disease ID: 122470, disease name: cornelia de lange syndrome, causative gene: NIPBL

HPO Term ID	Gender	Penetrance
HP:0002750	N/A	1.0
HP:0000664	N/A	0.98
HP:0000343	N/A	0.94
HP:0000219	N/A	0.94
HP:0002714	N/A	0.94
HP:0002983	N/A	0.93
HP:0002162	N/A	0.92
HP:0004691	N/A	0.86
HP:0000156	N/A	0.86
HP:0000463	N/A	0.85
HP:0001007	N/A	0.78
HP:0001612	N/A	0.74
HP:0000470	N/A	0.66
HP:0002987	N/A	0.64
HP:0008721	M	0.57
HP:0000545	N/A	0.57
HP:0000508	N/A	0.57
HP:0000639	N/A	0.57
HP:0000965	N/A	0.56
HP:0000954	N/A	0.51
HP:0001551	N/A	0.5
HP:0002557	N/A	0.5
HP:0000047	N/A	0.33
HP:0002020	N/A	0.3
HP:0001180	N/A	0.27
HP:0001249	N/A	0.25
HP:0000879	N/A	0.25
HP:0005815	N/A	0.25
HP:0003083	N/A	0.25

HP:0003997	N/A	0.25
HP:0001250	N/A	0.23
HP:0000588	N/A	0.075
HP:0000483	N/A	0.075
HP:0000648	N/A	0.075
HP:0001629	N/A	0.075
HP:0002984	N/A	0.075
HP:0000486	N/A	0.075
HP:0000520	N/A	0.075
HP:0001873	N/A	0.075
HP:0000059	N/A	0.075
HP:0002036	N/A	0.075
HP:0000776	N/A	0.075
HP:0000175	N/A	0.075
HP:0000482	N/A	0.075
HP:0002021	N/A	0.075
HP:0000453	N/A	0.075
HP:0000023	N/A	0.075
HP:0002020	N/A	0.075
HP:0005217	N/A	0.075
HP:0004785	N/A	0.075

OMIM: Disease ID: 131300, disease name: camurati-engelmann disease, causative gene: TGFB1

HPO Term ID	Gender	Penetrance
HP:0005791	N/A	0.9425
HP:0001533	N/A	0.9
HP:0009763	N/A	0.6847
HP:0002694	N/A	0.5423
HP:0004396	N/A	0.5
HP:0003202	N/A	0.5
HP:0003388	N/A	0.4444
HP:0002315	N/A	0.4090
HP:0001324	N/A	0.3913
HP:0001002	N/A	0.2127
HP:0000823	N/A	0.1666

OMIM: Disease ID: 142900, disease name: holt-oram syndrome, causative gene: TBX5

HPO Term ID	Gender	Penetrance
HP:0001631	N/A	0.4146
HP:0002984	N/A	0.3780
HP:0001629	N/A	0.1463
HP:0001643	N/A	0.075
HP:0002943	N/A	0.075
HP:0009751	N/A	0.075

OMIM: Disease ID: 147920, disease name: kabuki syndrome 1, causative gene: MLL2

HPO Term ID	Gender	Penetrance
HP:0000637	N/A	0.95
HP:0007655	N/A	0.9
HP:0000437	N/A	0.71
HP:0000175	N/A	0.44
HP:0000156	N/A	0.44
HP:0000252	N/A	0.25
HP:0000486	N/A	0.22
HP:0000592	N/A	0.21
HP:0001374	N/A	0.11
HP:0000508	N/A	0.11
HP:0001250	N/A	0.08

OMIM: Disease ID: 153400, disease name: lymphedema-distichiasis syndrome, causative gene: FOXC2

HPO Term ID	Gender	Penetrance
HP:0009743	N/A	1.0
HP:0003550	N/A	0.66
HP:0000508	N/A	0.075
HP:0000175	N/A	0.075
HP:0000465	N/A	0.075
HP:0000204	N/A	0.04

OMIM: Disease ID: 154500, disease name: treacher collins syndrome, causative gene: TCOF1

HPO Term ID	Gender	Penetrance
HP:0000272	N/A	0.89
HP:0000494	N/A	0.89
HP:0000347	N/A	0.78
HP:0000377	N/A	0.77
HP:0000652	N/A	0.69
HP:0000572	N/A	0.37
HP:0000185	N/A	0.32
HP:0000175	N/A	0.28
HP:0009554	N/A	0.26
HP:0009555	N/A	0.075
HP:0000160	N/A	0.075
HP:0000508	N/A	0.075
HP:0000197	N/A	0.075
HP:0000636	N/A	0.075
HP:0000486	N/A	0.075
HP:0000028	N/A	0.075
HP:0007678	N/A	0.075
HP:0000384	N/A	0.075
HP:0000453	N/A	0.075
HP:0002564	N/A	0.075
HP:0001249	N/A	0.05

OMIM: Disease ID: 161200, disease name: nail-patella syndrome, causative gene: LMX1B

HPO Term ID	Gender	Penetrance
HP:0001598	N/A	0.9831
HP:0001798	N/A	0.9831
HP:0001032	N/A	0.9579
HP:0001377	N/A	0.6958
HP:0009780	N/A	0.68
HP:0001763	N/A	0.6440
HP:0009781	N/A	0.5378
HP:0000767	N/A	0.3589
HP:0002650	N/A	0.2285
HP:0001762	N/A	0.1885
HP:0009760	N/A	0.1219
HP:0000501	N/A	0.0963
HP:0009788	N/A	0.075
HP:0009785	N/A	0.075
HP:0009783	N/A	0.075
HP:0005255	N/A	0.075

OMIM: Disease ID: 162200, disease name: neurofibromatosis, type i, causative gene: NF1

HPO Term ID	Gender	Penetrance
HP:0009737	N/A	0.95
HP:0001328	N/A	0.3
HP:0009732	N/A	0.3
HP:0002650	N/A	0.05
HP:0009736	N/A	0.04
HP:0001920	N/A	0.015
HP:0002521	N/A	0.015
HP:0009734	N/A	0.015
HP:0002857	N/A	0.015
HP:0002410	N/A	0.015
HP:0002666	N/A	0.01
HP:0002858	N/A	0.01
HP:0000501	N/A	0.007

OMIM: Disease ID: 175700, disease name: greig cephalopolysyndactyly syndrome, causative gene: GLI3

HPO Term ID	Gender	Penetrance
HP:0001162	N/A	0.78
HP:0000348	N/A	0.7
HP:0000256	N/A	0.52
HP:0000316	N/A	0.25
HP:0001256	N/A	0.075
HP:0001537	N/A	0.075
HP:0000494	N/A	0.075
HP:0001274	N/A	0.075
HP:0004303	N/A	0.075
HP:0005616	N/A	0.075
HP:0001830	N/A	0.075
HP:0001007	N/A	0.075
HP:0000238	N/A	0.075
HP:0000023	N/A	0.075
HP:0001250	N/A	0.075
HP:0001363	N/A	0.075
HP:0000047	N/A	0.075
HP:0000028	N/A	0.075

OMIM: Disease ID: 176450, disease name: currarino syndrome, causative gene: MNX1

HPO Term ID	Gender	Penetrance
HP:0009790	N/A	0.75
HP:0007293	N/A	0.4975
HP:0000143	N/A	0.33
HP:0009793	N/A	0.33
HP:0000010	N/A	0.33
HP:0009791	N/A	0.22
HP:0004796	N/A	0.1609
HP:0002144	N/A	0.1414
HP:0001287	N/A	0.1121
HP:0009789	N/A	0.1121
HP:0000076	N/A	0.075
HP:0000813	N/A	0.075

OMIM: Disease ID: 180849, disease name: rubinstein-taybi syndrome 1, causative gene: CREBBP

HPO Term ID	Gender	Penetrance
HP:0001249	N/A	1.0
HP:0000444	N/A	0.9
HP:0000494	N/A	0.88
HP:0000527	N/A	0.87
HP:0002317	N/A	0.85
HP:0000377	N/A	0.84
HP:0000028	M	0.78
HP:0000574	N/A	0.76
HP:0001007	N/A	0.75
HP:0002750	N/A	0.74
HP:0001763	N/A	0.72
HP:0004411	N/A	0.71
HP:0000486	N/A	0.69
HP:0002353	N/A	0.57
HP:0000286	N/A	0.55
HP:0003298	N/A	0.47
HP:0000579	N/A	0.43
HP:0002650	N/A	0.42
HP:0002162	N/A	0.42
HP:0000260	N/A	0.41
HP:0000252	N/A	0.35
HP:0001631	N/A	0.33
HP:0001629	N/A	0.33
HP:0001643	N/A	0.33
HP:0008107	N/A	0.33
HP:0001135	N/A	0.31
HP:0005306	N/A	0.25
HP:0000294	N/A	0.24
HP:0000270	N/A	0.24

HP:0001250	N/A	0.23
HP:0000663	N/A	0.22
HP:0002236	N/A	0.2
HP:0000954	N/A	0.075
HP:0002700	N/A	0.075
HP:0000508	N/A	0.075
HP:0000049	M	0.075
HP:0000047	M	0.075
HP:0000501	N/A	0.075
HP:0002697	N/A	0.075
HP:0002999	N/A	0.075
HP:0000518	N/A	0.075

OMIM: Disease ID: 182212, disease name: shprintzen-goldberg craniosynostosis syndrome, causative gene: SKI

HPO Term ID	Gender	Penetrance
HP:0001166	N/A	0.9189
HP:0000368	N/A	0.8648
HP:0000494	N/A	0.8378
HP:0000156	N/A	0.8108
HP:0000268	N/A	0.7567
HP:0000316	N/A	0.7567
HP:0002650	N/A	0.6216
HP:0000377	N/A	0.5405
HP:0000023	N/A	0.5135
HP:0001363	N/A	0.4864
HP:0000486	N/A	0.4594
HP:0000508	N/A	0.4324
HP:0000327	N/A	0.4324
HP:0001537	N/A	0.3513
HP:0001634	N/A	0.3513
HP:0001762	N/A	0.3513
HP:0000938	N/A	0.2702
HP:0000260	N/A	0.2432
HP:0000586	N/A	0.2432
HP:0000545	N/A	0.2307
HP:0000252	N/A	0.1621
HP:0002870	N/A	0.1621
HP:0000895	N/A	0.1621
HP:0003083	N/A	0.1351
HP:0009023	N/A	0.1351
HP:0005815	N/A	0.1351
HP:0000405	N/A	0.1351
HP:0002857	N/A	0.1081
HP:0003016	N/A	0.1081

HP:0002020	N/A	0.1081
HP:0000028	N/A	0.1081

OMIM: Disease ID: 191100, disease name: tuberous sclerosis 1, causative gene: TSC1

HPO Term ID	Gender	Penetrance
HP:0009716	N/A	0.95
HP:0000717	N/A	0.93
HP:0009720	N/A	0.85
HP:0009719	N/A	0.82
HP:0001249	N/A	0.53
HP:0006772	N/A	0.52
HP:0009729	N/A	0.5
HP:0001482	N/A	0.5
HP:0009554	N/A	0.5
HP:0009722	N/A	0.48
HP:0009724	N/A	0.32
HP:0009721	N/A	0.31
HP:0009727	N/A	0.13
HP:0009717	N/A	0.1
HP:0005584	N/A	0.05

OMIM: Disease ID: 193700, disease name: arthrogryposis, distal, type 2a, causative gene: MYH3

HPO Term ID	Gender	Penetrance
HP:0000346	N/A	1.0
HP:0002751	N/A	0.84
HP:0003273	N/A	0.73
HP:0001762	N/A	0.59
HP:0000252	N/A	0.44
HP:0000506	N/A	0.25
HP:0000490	N/A	0.25
HP:0000343	N/A	0.25
HP:0000486	N/A	0.25
HP:0000581	N/A	0.25
HP:0001838	N/A	0.25
HP:0000431	N/A	0.25
HP:0000430	N/A	0.25
HP:0003044	N/A	0.25
HP:0001181	N/A	0.25
HP:0000286	N/A	0.25
HP:0000023	N/A	0.25
HP:0001611	N/A	0.25
HP:0001250	N/A	0.19
HP:0002365	N/A	0.075
HP:0002365	N/A	0.075
HP:0003298	N/A	0.075
HP:0000508	N/A	0.075
HP:0000470	N/A	0.075
HP:0002827	N/A	0.075
HP:0001518	N/A	0.075

OMIM: Disease ID: 216550, disease name: cohen syndrome, causative gene: VPS13B

HPO Term ID	Gender	Penetrance
HP:0001249	N/A	1.0
HP:0001763	N/A	0.97
HP:0000252	N/A	0.9032
HP:0001182	N/A	0.78
HP:0001319	N/A	0.56
HP:0000545	N/A	0.5
HP:0000426	N/A	0.5
HP:0000444	N/A	0.5
HP:0000322	N/A	0.5
HP:0001135	N/A	0.5
HP:0000574	N/A	0.5
HP:0004283	N/A	0.5
HP:0000675	N/A	0.5
HP:0000494	N/A	0.5
HP:0000823	N/A	0.4
HP:0000505	N/A	0.35
HP:0001601	N/A	0.33
HP:0001518	N/A	0.33

OMIM: Disease ID: 218600, disease name: baller-gerold syndrome, causative gene: RECQL4

HPO Term ID	Gender	Penetrance
HP:0003031	N/A	0.68
HP:0000368	N/A	0.64
HP:0001249	N/A	0.5
HP:0000160	N/A	0.32
HP:0000494	N/A	0.32
HP:0000426	N/A	0.32
HP:0004425	N/A	0.27
HP:0003298	N/A	0.075
HP:0000486	N/A	0.075
HP:0000405	N/A	0.075
HP:0002996	N/A	0.075
HP:0000175	N/A	0.075
HP:0000545	N/A	0.075
HP:0000193	N/A	0.075
HP:0000902	N/A	0.075
HP:0006467	N/A	0.075
HP:0002126	N/A	0.075
HP:0000238	N/A	0.075
HP:0000286	N/A	0.075
HP:0001250	N/A	0.075
HP:0005201	N/A	0.075
HP:0000452	N/A	0.075
HP:0003065	N/A	0.075
HP:0005792	N/A	0.075
HP:0000430	N/A	0.075
HP:0000648	N/A	0.075
HP:0001274	N/A	0.075

OMIM: Disease ID: 225750, disease name: aicardi-goutieres syndrome, causative gene: TREX1

HPO Term ID	Gender	Penetrance
HP:0002062	N/A	0.9855
HP:0009709	N/A	0.9393
HP:0002135	N/A	0.9275
HP:0009704	N/A	0.7846
HP:0002071	N/A	0.6086
HP:0001433	N/A	0.5652
HP:0000639	N/A	0.5238
HP:0002448	N/A	0.5
HP:0002421	N/A	0.5
HP:0001250	N/A	0.5
HP:0007321	N/A	0.5
HP:0001332	N/A	0.5
HP:0000486	N/A	0.2857
HP:0009710	N/A	0.2380
HP:0004394	N/A	0.1594
HP:0001945	N/A	0.1594

OMIM: Disease ID: 235730, disease name: mowat-wilson syndrome, causative gene: ZEB2

HPO Term ID	Gender	Penetrance
HP:0001250	N/A	0.9
HP:0000252	N/A	0.84
HP:0000307	N/A	0.5
HP:0005274	N/A	0.5
HP:0000494	N/A	0.5
HP:0000508	N/A	0.5
HP:0000490	N/A	0.5
HP:0002019	N/A	0.5
HP:0000316	N/A	0.5
HP:0000431	N/A	0.5
HP:0000378	N/A	0.5
HP:0000612	N/A	0.5
HP:0000565	N/A	0.5
HP:0001274	N/A	0.42
HP:0003720	N/A	0.33
HP:0000048	M	0.33
HP:0000028	M	0.33
HP:0000047	M	0.33
HP:0000176	N/A	0.075

OMIM: Disease ID: 236670, disease name: muscular dystrophy-dystroglycanopathy, causative gene: POMT1

HPO Term ID	Gender	Penetrance
HP:0007260	N/A	1.0
HP:0007901	N/A	1.0
HP:0003741	N/A	1.0
HP:0002189	N/A	0.95
HP:0000238	N/A	0.53
HP:0000568	N/A	0.53
HP:0001305	N/A	0.53
HP:0002803	N/A	0.43
HP:0006891	N/A	0.25
HP:0000541	N/A	0.25
HP:0007973	N/A	0.25
HP:0001274	N/A	0.25
HP:0002079	N/A	0.25
HP:0006891	N/A	0.25
HP:0002085	N/A	0.24
HP:0000252	N/A	0.16
HP:0000413	N/A	0.075
HP:0000485	N/A	0.075
HP:0000110	N/A	0.075

OMIM: Disease ID: 236680, disease name: hydrolethalus syndrome 1, causative gene: HYLS1

HPO Term ID	Gender	Penetrance
HP:0006882	N/A	0.92
HP:0001561	N/A	0.92
HP:0000568	N/A	0.86
HP:0001162	N/A	0.77
HP:0009752	N/A	0.76
HP:0000377	N/A	0.7
HP:0002777	N/A	0.57
HP:0008749	N/A	0.57
HP:0000175	N/A	0.55
HP:0001762	N/A	0.52
HP:0001674	N/A	0.48
HP:0001629	N/A	0.48
HP:0000047	M	0.33
HP:0000136	F	0.33
HP:0000126	N/A	0.16

OMIM: Disease ID: 243800, disease name: johanson-blizzard syndrome, causative gene: UBR1

HPO Term ID	Gender	Penetrance
HP:0002024	N/A	1.0
HP:0000430	N/A	1.0
HP:0002209	N/A	0.96
HP:0002236	N/A	0.96
HP:0006334	N/A	0.9
HP:0006349	N/A	0.9
HP:0001249	N/A	0.67
HP:0001545	N/A	0.4
HP:0000821	N/A	0.3
HP:0000126	F	0.25
HP:0001153	F	0.25
HP:0000126	M	0.25
HP:0000028	M	0.25
HP:0000054	M	0.25
HP:0000047	M	0.25
HP:0000143	N/A	0.18
HP:0000954	N/A	0.075
HP:0000819	N/A	0.075
HP:0002557	N/A	0.075
HP:0001362	N/A	0.075
HP:0000486	N/A	0.075
HP:0002253	N/A	0.075
HP:0003362	N/A	0.075
HP:0001696	N/A	0.075
HP:0000957	N/A	0.075

OMIM: Disease ID: 265000, disease name: multiple pterygium syndrome, escobar variant, causative gene: CHRNG

HPO Term ID	Gender	Penetrance
HP:0002650	N/A	1.0
HP:0001836	N/A	1.0
HP:0006446	N/A	1.0
HP:0005617	N/A	1.0
HP:0000508	N/A	0.8181
HP:0000494	N/A	0.8181
HP:0008729	F	0.8
HP:0002643	N/A	0.7272
HP:0001762	N/A	0.7272
HP:0002714	N/A	0.7272
HP:0002808	N/A	0.7
HP:0000028	M	0.6666
HP:0009759	N/A	0.6363
HP:0009756	N/A	0.5454
HP:0000286	N/A	0.5454
HP:0000160	N/A	0.5
HP:0000343	N/A	0.4545
HP:0003083	N/A	0.4285
HP:0001060	N/A	0.3636
HP:0002949	N/A	0.3
HP:0009761	N/A	0.3
HP:0009760	N/A	0.2727
HP:0000405	N/A	0.2727
HP:0000902	N/A	0.2222
HP:0009757	N/A	0.1818
HP:0000175	N/A	0.1818

OMIM: Disease ID: 270400, disease name: smith-lemli-opitz syndrome, causative gene: DHCR7

HPO Term ID	Gender	Penetrance
HP:0004691	N/A	0.99
HP:0001249	N/A	0.9512
HP:0000252	N/A	0.8414
HP:0001508	N/A	0.8170
HP:0000463	N/A	0.75
HP:0000508	N/A	0.7012
HP:0000343	N/A	0.5
HP:0002079	N/A	0.5
HP:0000047	M	0.5
HP:0007165	N/A	0.5
HP:0000187	N/A	0.5
HP:0001830	N/A	0.4817
HP:0001162	N/A	0.4817
HP:0000175	N/A	0.4695
HP:0000104	N/A	0.33
HP:0000518	N/A	0.2195
HP:0001631	N/A	0.2
HP:0001643	N/A	0.18
HP:0002021	N/A	0.1402
HP:0001629	N/A	0.1
HP:0000316	N/A	0.075
HP:0000286	N/A	0.075

OMIM: Disease ID: 303600, disease name: coffin-lowry syndrome, causative gene: RPS6KA3

HPO Term ID	Gender	Penetrance
HP:0001249	N/A	1.0
HP:0001169	N/A	1.0
HP:0001182	N/A	1.0
HP:0000445	N/A	1.0
HP:0009746	N/A	1.0
HP:0000316	N/A	1.0
HP:0000506	N/A	1.0
HP:0000280	N/A	1.0
HP:0006129	N/A	0.96
HP:0000954	N/A	0.9411
HP:0000494	N/A	0.8518
HP:0000767	N/A	0.8
HP:0000768	N/A	0.8
HP:0002750	N/A	0.7894
HP:0002684	N/A	0.6363
HP:0001250	N/A	0.4705

OMIM: Disease ID: 305400, disease name: aarskog-scott syndrome, causative gene: FGD1

HPO Term ID	Gender	Penetrance
HP:0000316	N/A	0.89
HP:0000289	N/A	0.8461
HP:0009748	N/A	0.8252
HP:0001174	N/A	0.8181
HP:0001156	N/A	0.8181
HP:0002055	N/A	0.8080
HP:0000327	N/A	0.8076
HP:0000463	N/A	0.7924
HP:0000049	N/A	0.7708
HP:0000349	N/A	0.7647
HP:0006158	N/A	0.7540
HP:0000954	N/A	0.6909
HP:0000431	N/A	0.6666
HP:0001544	N/A	0.6666
HP:0000028	N/A	0.6382
HP:0000023	N/A	0.6170
HP:0000494	N/A	0.5308
HP:0000508	N/A	0.5061
HP:0000767	N/A	0.4594

OMIM: Disease ID: 305450, disease name: opitz-kaveggia syndrome, causative gene: MED12

HPO Term ID	Gender	Penetrance
HP:0001249	N/A	0.97
HP:0002236	N/A	0.91
HP:0001270	N/A	0.9
HP:0000494	N/A	0.85
HP:0000316	N/A	0.83
HP:0005833	N/A	0.81
HP:0000260	N/A	0.77
HP:0005490	N/A	0.74
HP:0001250	N/A	0.7
HP:0002019	N/A	0.69
HP:0000954	N/A	0.6
HP:0000286	N/A	0.56
HP:0001159	N/A	0.54
HP:0000486	N/A	0.52
HP:0000179	N/A	0.44
HP:0002025	N/A	0.38
HP:0001150	N/A	0.38
HP:0001545	N/A	0.38
HP:0000028	N/A	0.36
HP:0000960	N/A	0.25
HP:0002828	N/A	0.25
HP:0009762	N/A	0.25
HP:0008070	N/A	0.25
HP:0002213	N/A	0.25
HP:0001620	N/A	0.075
HP:0001171	N/A	0.075
HP:0000470	N/A	0.075
HP:0000175	N/A	0.075
HP:0002021	N/A	0.075

HP:0000453	N/A	0.075
HP:0000047	N/A	0.075
